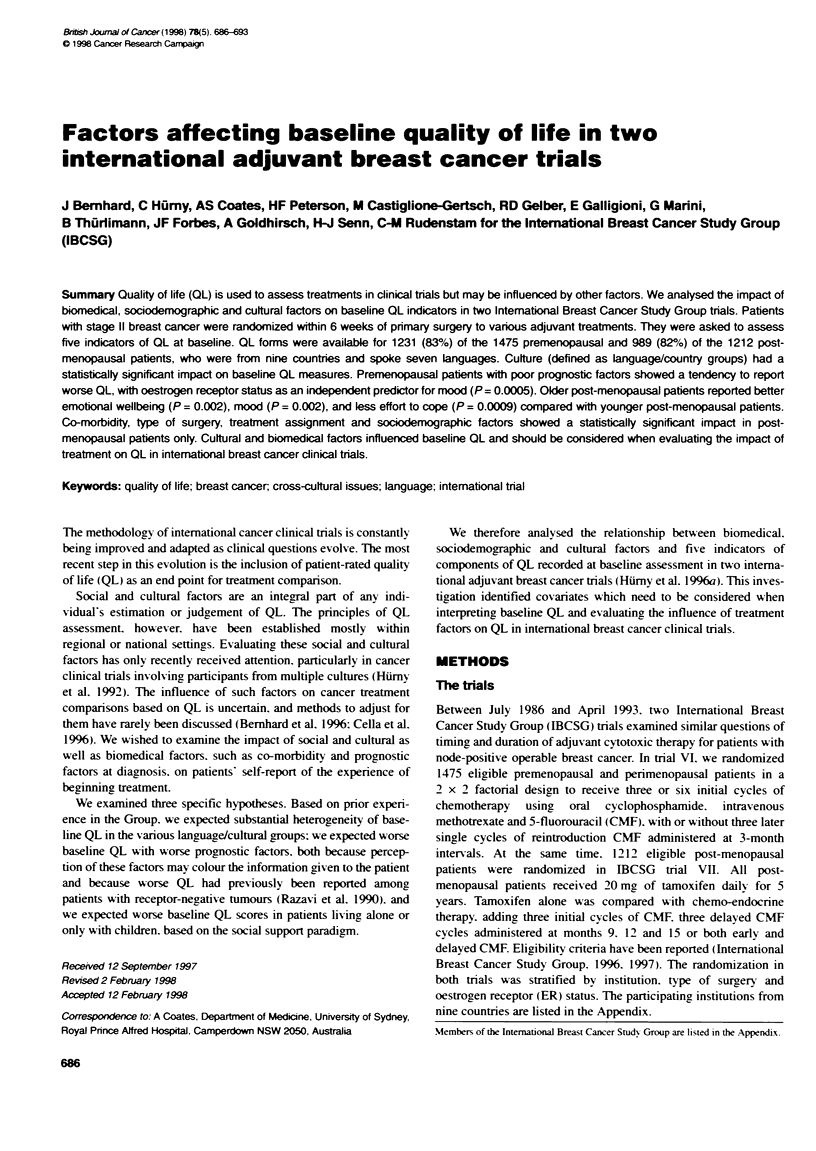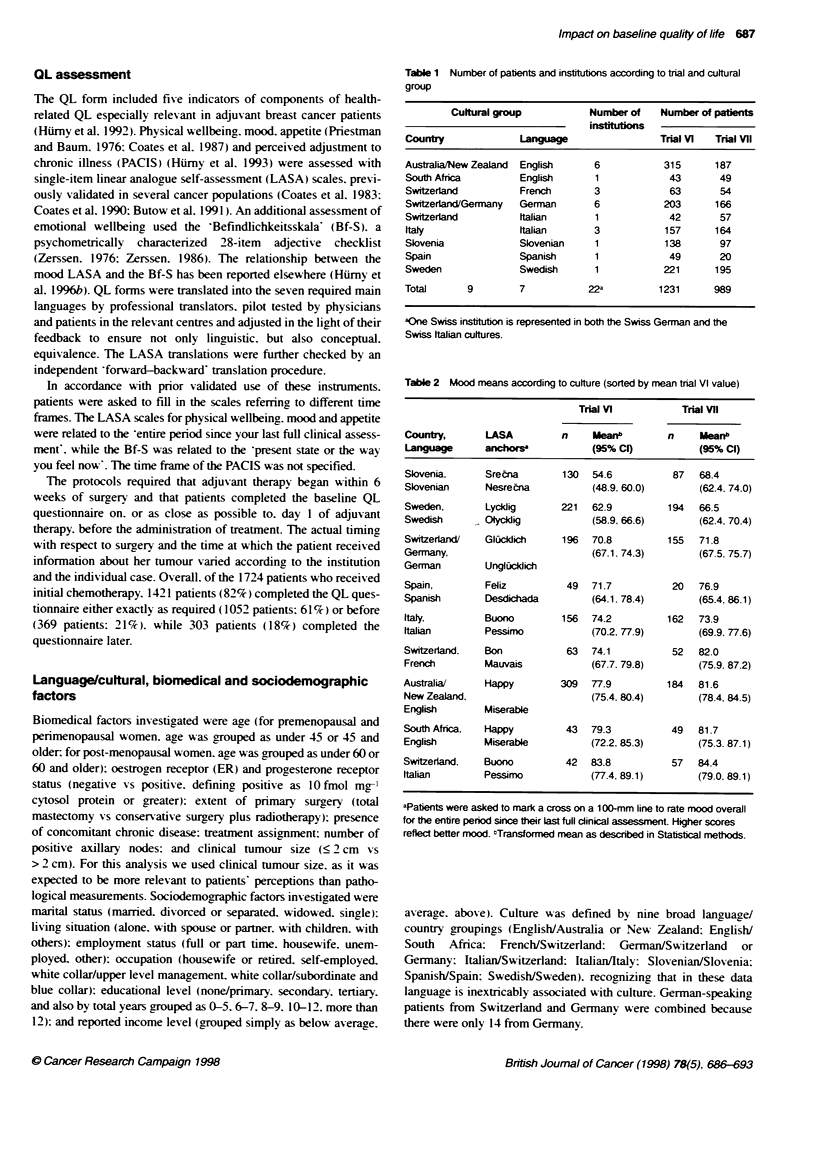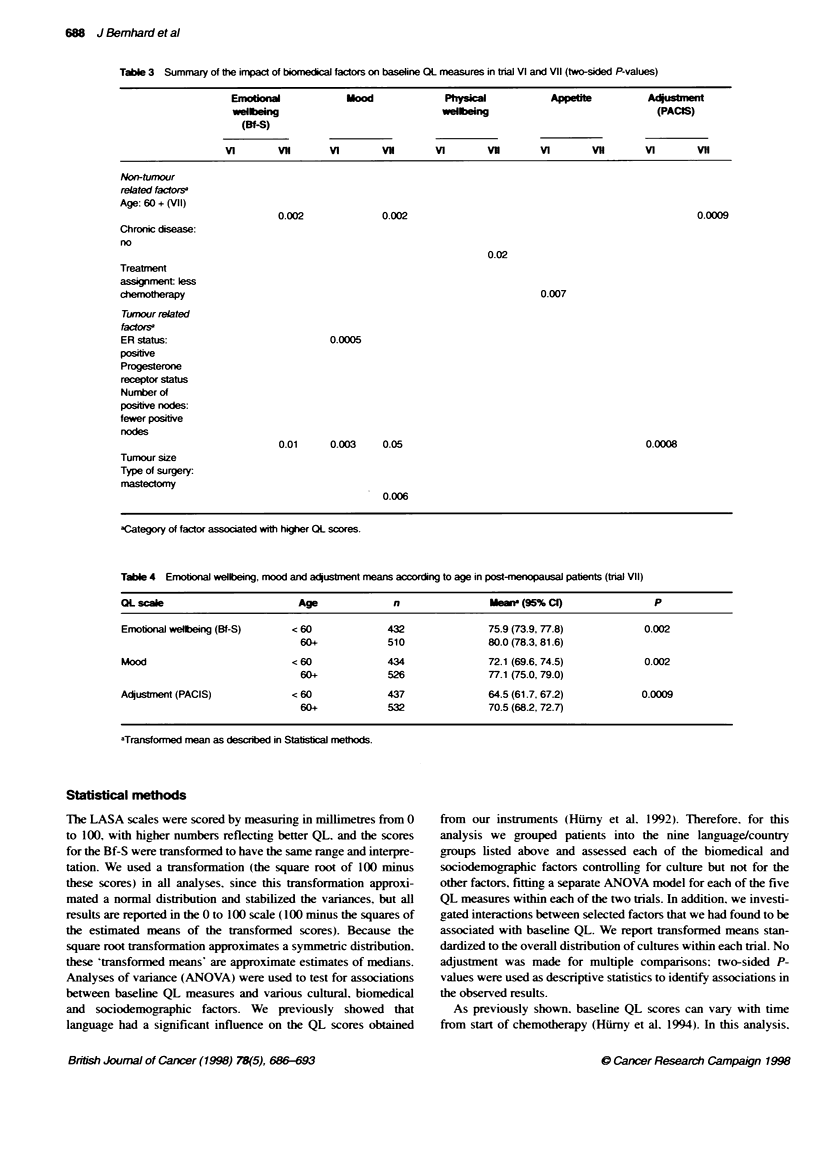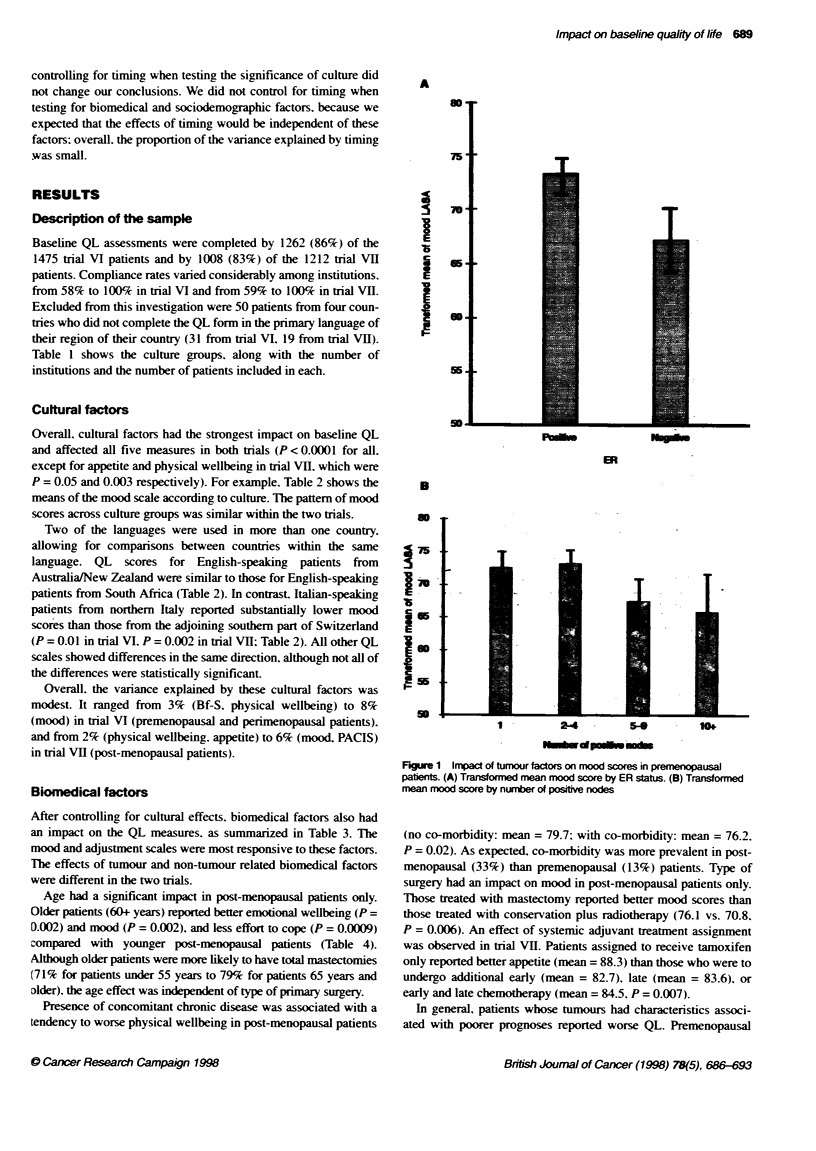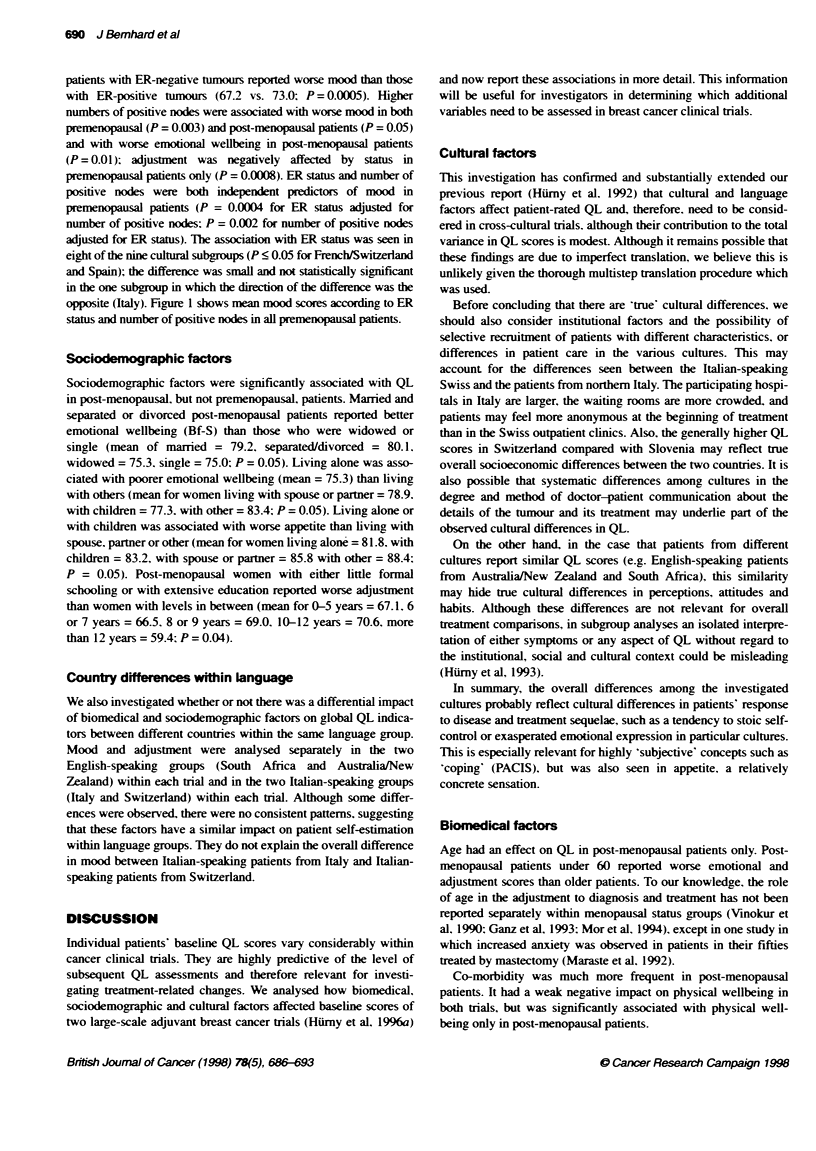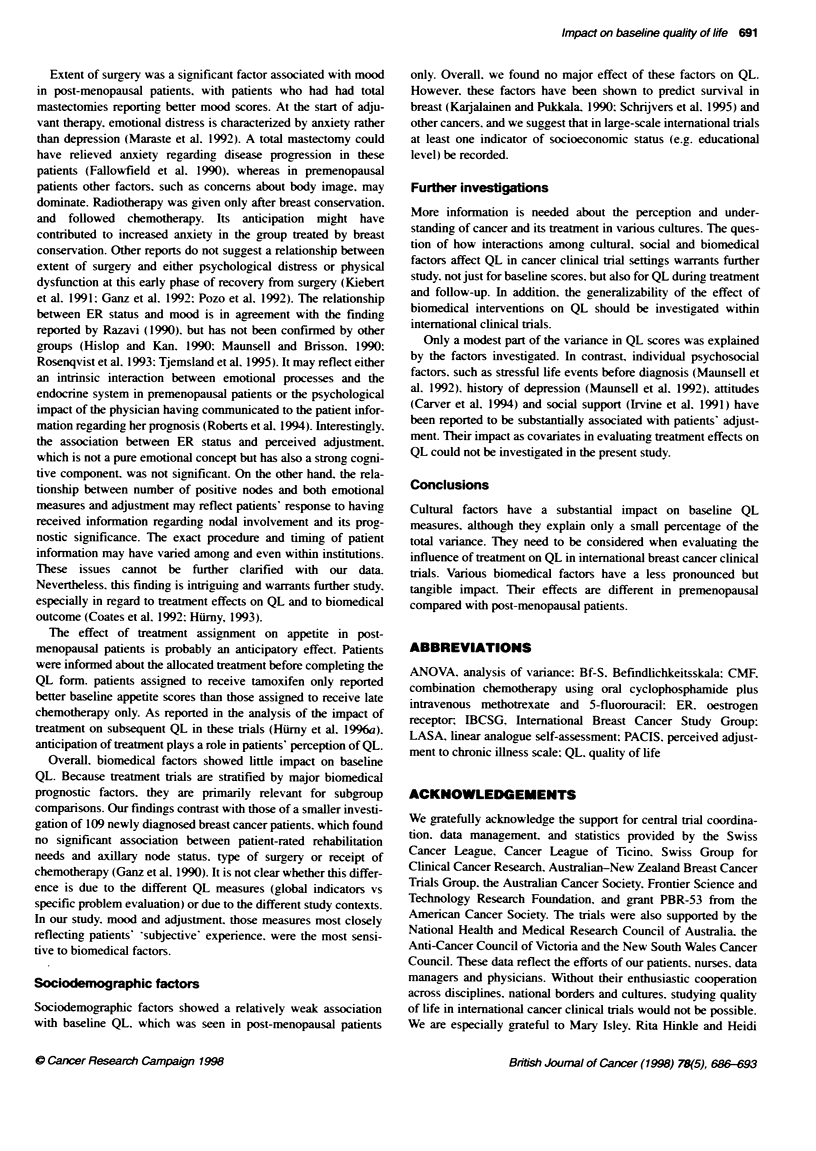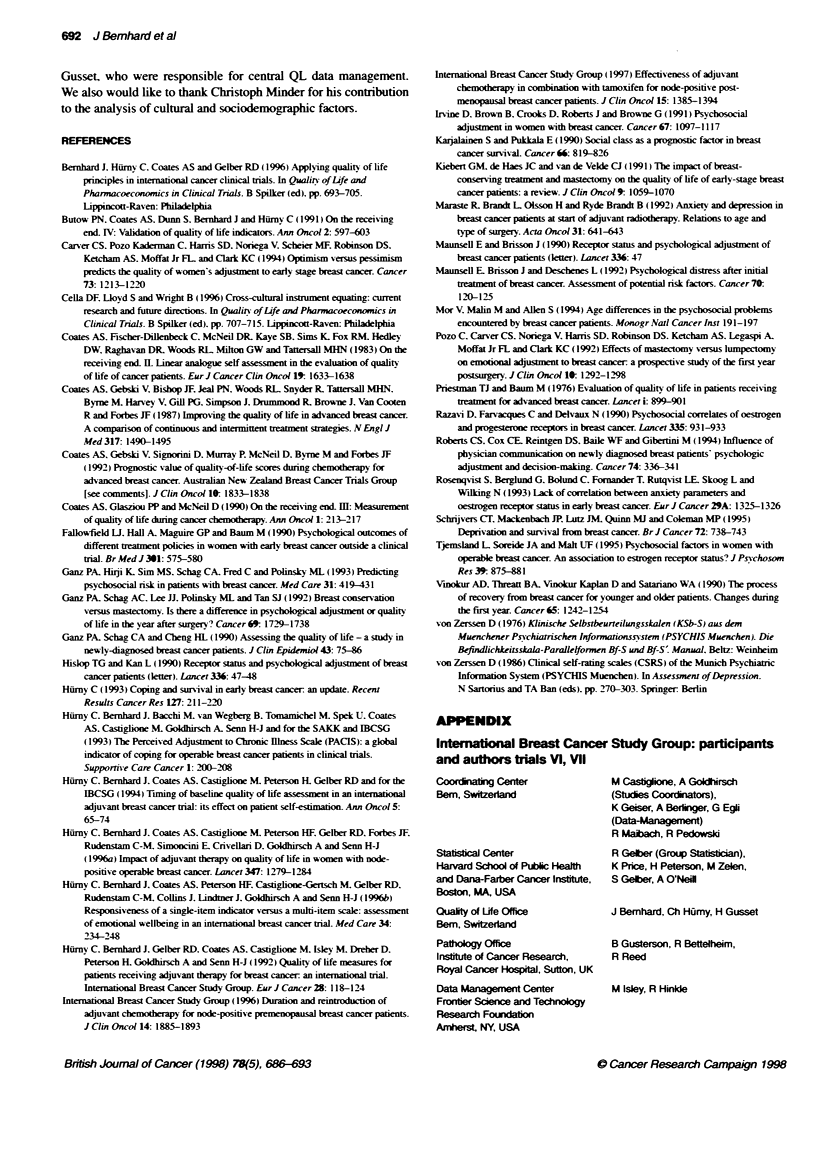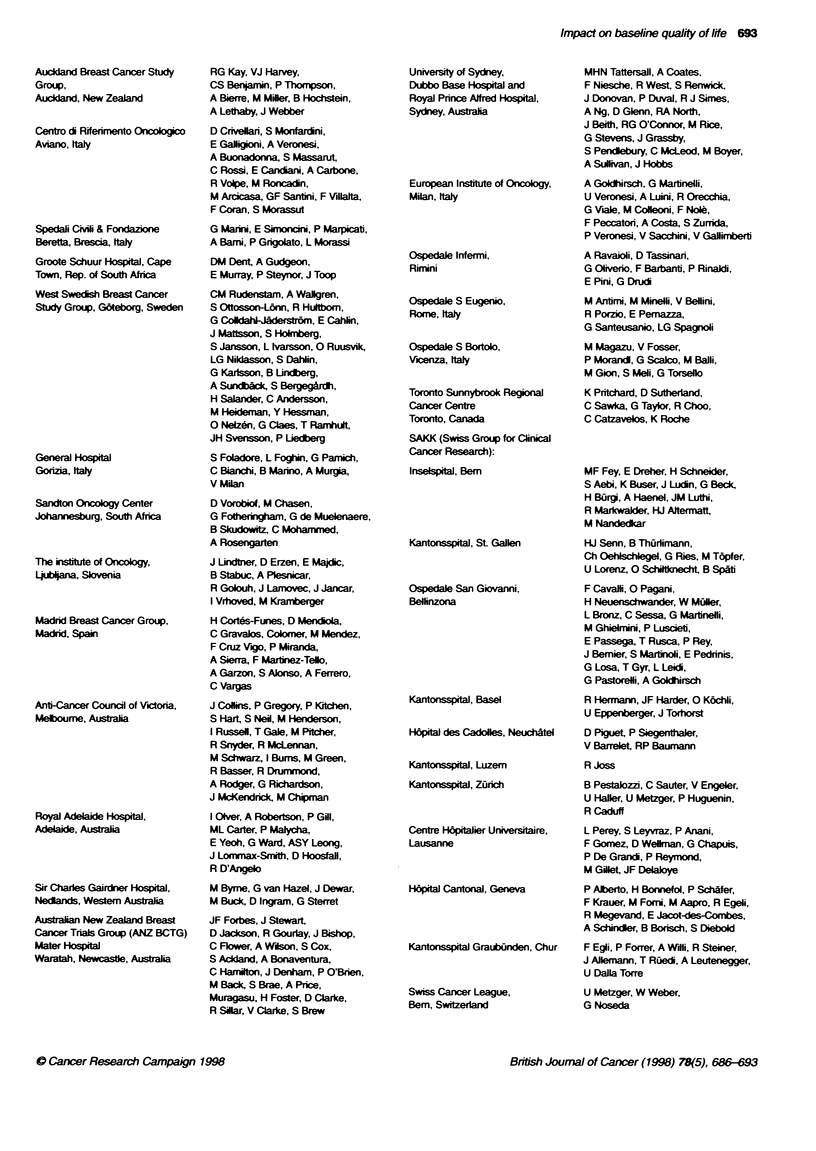# Factors affecting baseline quality of life in two international adjuvant breast cancer trials. International Breast Cancer Study Group (IBCSG).

**DOI:** 10.1038/bjc.1998.561

**Published:** 1998-09

**Authors:** J. Bernhard, C. Hürny, A. S. Coates, H. F. Peterson, M. Castiglione-Gertsch, R. D. Gelber, E. Galligioni, G. Marini, B. Thürlimann, J. F. Forbes, A. Goldhirsch, H. J. Senn, C. M. Rudenstam

## Abstract

Quality of life (QL) is used to assess treatments in clinical trials but may be influenced by other factors. We analysed the impact of biomedical, sociodemographic and cultural factors on baseline QL indicators in two International Breast Cancer Study Group trials. Patients with stage II breast cancer were randomized within 6 weeks of primary surgery to various adjuvant treatments. They were asked to assess five indicators of QL at baseline. QL forms were available for 1231 (83%) of the 1475 premenopausal and 989 (82%) of the 1212 post-menopausal patients, who were from nine countries and spoke seven languages. Culture (defined as language/country groups) had a statistically significant impact on baseline QL measures. Premenopausal patients with poor prognostic factors showed a tendency to report worse QL, with oestrogen receptor status as an independent predictor for mood (P = 0.0005). Older post-menopausal patients reported better emotional wellbeing (P = 0.002), mood (P = 0.002), and less effort to cope (P = 0.0009) compared with younger post-menopausal patients. Co-morbidity, type of surgery, treatment assignment and sociodemographic factors showed a statistically significant impact in post-menopausal patients only. Cultural and biomedical factors influenced baseline QL and should be considered when evaluating the impact of treatment on QL in international breast cancer clinical trials.


British Journal of Cancer (1998) 78(5), 686–693
© 1998 Cancer Research Campaign

### Factors affecting baseline quality of life in two international adjuvant breast cancer trials

J Bernhard, C Hürny, AS Coates, HF Peterson, M Castiglione-Gertsch, RD Gelber, E Galligioni, G Marini, B Thürlimann, JF Forbes, A Goldhirsch, H-J Senn, C-M Rudenstam for the International Breast Cancer Study Group (IBCSG)

**Summary** Quality of life (QL) is used to assess treatments in clinical trials but may be influenced by other factors. We analysed the impact of biomedical, sociodemographic and cultural factors on baseline QL indicators in two International Breast Cancer Study Group trials. Patients with stage II breast cancer were randomized within 6 weeks of primary surgery to various adjuvant treatments. They were asked to assess five indicators of QL at baseline. QL forms were available for 1231 (83%) of the 1475 premenopausal and 989 (82%) of the 1212 post-menopausal patients, who were from nine countries and spoke seven languages. Culture (defined as language/country groups) had a statistically significant impact on baseline QL measures. Premenopausal patients with poor prognostic factors showed a tendency to report worse QL, with oestrogen receptor status as an independent predictor for mood ($P = 0.0005$). Older post-menopausal patients reported better emotional wellbeing ($P = 0.002$), mood ($P = 0.002$), and less effort to cope ($P = 0.0009$) compared with younger post-menopausal patients. Co-morbidity, type of surgery, treatment assignment and sociodemographic factors showed a statistically significant impact in post-menopausal patients only. Cultural and biomedical factors influenced baseline QL and should be considered when evaluating the impact of treatment on QL in international breast cancer clinical trials.

**Keywords:** quality of life; breast cancer; cross-cultural issues; language; international trial

The methodology of international cancer clinical trials is constantly being improved and adapted as clinical questions evolve. The most recent step in this evolution is the inclusion of patient-rated quality of life (QL) as an end point for treatment comparison.

Social and cultural factors are an integral part of any individual's estimation or judgement of QL. The principles of QL assessment, however, have been established mostly within regional or national settings. Evaluating these social and cultural factors has only recently received attention, particularly in cancer clinical trials involving participants from multiple cultures (Hürny et al, 1992). The influence of such factors on cancer treatment comparisons based on QL is uncertain, and methods to adjust for them have rarely been discussed (Bernhard et al, 1996; Cella et al, 1996). We wished to examine the impact of social and cultural as well as biomedical factors, such as co-morbidity and prognostic factors at diagnosis, on patients' self-report of the experience of beginning treatment.

We examined three specific hypotheses. Based on prior experience in the Group, we expected substantial heterogeneity of baseline QL in the various language/cultural groups; we expected worse baseline QL with worse prognostic factors, both because perception of these factors may colour the information given to the patient and because worse QL had previously been reported among patients with receptor-negative tumours (Razavi et al, 1990), and we expected worse baseline QL scores in patients living alone or only with children, based on the social support paradigm.

*Received 12 September 1997*
*Revised 2 February 1998*
*Accepted 12 February 1998*

*Correspondence to:* A Coates, Department of Medicine, University of Sydney, Royal Prince Alfred Hospital, Camperdown NSW 2050, Australia

We therefore analysed the relationship between biomedical, sociodemographic and cultural factors and five indicators of components of QL recorded at baseline assessment in two international adjuvant breast cancer trials (Hürny et al, 1996*a*). This investigation identified covariates which need to be considered when interpreting baseline QL and evaluating the influence of treatment factors on QL in international breast cancer clinical trials.

### METHODS

### The trials

Between July 1986 and April 1993, two International Breast Cancer Study Group (IBCSG) trials examined similar questions of timing and duration of adjuvant cytotoxic therapy for patients with node-positive operable breast cancer. In trial VI, we randomized 1475 eligible premenopausal and perimenopausal patients in a 2 × 2 factorial design to receive three or six initial cycles of chemotherapy using oral cyclophosphamide, intravenous methotrexate and 5-fluorouracil (CMF), with or without three later single cycles of reintroduction CMF administered at 3-month intervals. At the same time, 1212 eligible post-menopausal patients were randomized in IBCSG trial VII. All post-menopausal patients received 20 mg of tamoxifen daily for 5 years. Tamoxifen alone was compared with chemo-endocrine therapy, adding three initial cycles of CMF, three delayed CMF cycles administered at months 9, 12 and 15 or both early and delayed CMF. Eligibility criteria have been reported (International Breast Cancer Study Group, 1996, 1997). The randomization in both trials was stratified by institution, type of surgery and oestrogen receptor (ER) status. The participating institutions from nine countries are listed in the Appendix.

Members of the International Breast Cancer Study Group are listed in the Appendix.

### QL assessment

The QL form included five indicators of components of health-related QL especially relevant in adjuvant breast cancer patients (Hürny et al. 1992). Physical wellbeing, mood, appetite (Priestman and Baum, 1976; Coates et al. 1987) and perceived adjustment to chronic illness (PACIS) (Hürny et al. 1993) were assessed with single-item linear analogue self-assessment (LASA) scales, previously validated in several cancer populations (Coates et al. 1983; Coates et al. 1990; Butow et al. 1991). An additional assessment of emotional wellbeing used the 'Befindlichkeitsskala' (Bf-S), a psychometrically characterized 28-item adjective checklist (Zerssen, 1976; Zerssen, 1986). The relationship between the mood LASA and the Bf-S has been reported elsewhere (Hürny et al. 1996*b*). QL forms were translated into the seven required main languages by professional translators, pilot tested by physicians and patients in the relevant centres and adjusted in the light of their feedback to ensure not only linguistic, but also conceptual, equivalence. The LASA translations were further checked by an independent 'forward–backward' translation procedure.

In accordance with prior validated use of these instruments, patients were asked to fill in the scales referring to different time frames. The LASA scales for physical wellbeing, mood and appetite were related to the 'entire period since your last full clinical assessment', while the Bf-S was related to the 'present state or the way you feel now'. The time frame of the PACIS was not specified.

The protocols required that adjuvant therapy began within 6 weeks of surgery and that patients completed the baseline QL questionnaire on, or as close as possible to, day 1 of adjuvant therapy, before the administration of treatment. The actual timing with respect to surgery and the time at which the patient received information about her tumour varied according to the institution and the individual case. Overall, of the 1724 patients who received initial chemotherapy, 1421 patients (82%) completed the QL questionnaire either exactly as required (1052 patients; 61%) or before (369 patients; 21%), while 303 patients (18%) completed the questionnaire later.

### Language/cultural, biomedical and sociodemographic factors

Biomedical factors investigated were age (for premenopausal and perimenopausal women, age was grouped as under 45 or 45 and older; for post-menopausal women, age was grouped as under 60 or 60 and older); oestrogen receptor (ER) and progesterone receptor status (negative vs positive, defining positive as 10 fmol $mg^{-1}$ cytosol protein or greater); extent of primary surgery (total mastectomy vs conservative surgery plus radiotherapy); presence of concomitant chronic disease; treatment assignment; number of positive axillary nodes; and clinical tumour size ($\leq 2$ cm vs $> 2$ cm). For this analysis we used clinical tumour size, as it was expected to be more relevant to patients' perceptions than pathological measurements. Sociodemographic factors investigated were marital status (married, divorced or separated, widowed, single); living situation (alone, with spouse or partner, with children, with others); employment status (full or part time, housewife, unemployed, other); occupation (housewife or retired, self-employed, white collar/upper level management, white collar/subordinate and blue collar); educational level (none/primary, secondary, tertiary, and also by total years grouped as 0–5, 6–7, 8–9, 10–12, more than 12); and reported income level (grouped simply as below average, average, above). Culture was defined by nine broad language/country groupings (English/Australia or New Zealand; English/South Africa; French/Switzerland; German/Switzerland or Germany; Italian/Switzerland; Italian/Italy; Slovenian/Slovenia; Spanish/Spain; Swedish/Sweden), recognizing that in these data language is inextricably associated with culture. German-speaking patients from Switzerland and Germany were combined because there were only 14 from Germany.

**Table 1** Number of patients and institutions according to trial and cultural group

| Cultural group | | Number of institutions | Number of patients | |
|---|---|---|---|---|
| Country | Language | | Trial VI | Trial VII |
| Australia/New Zealand | English | 6 | 315 | 187 |
| South Africa | English | 1 | 43 | 49 |
| Switzerland | French | 3 | 63 | 54 |
| Switzerland/Germany | German | 6 | 203 | 166 |
| Switzerland | Italian | 1 | 42 | 57 |
| Italy | Italian | 3 | 157 | 164 |
| Slovenia | Slovenian | 1 | 138 | 97 |
| Spain | Spanish | 1 | 49 | 20 |
| Sweden | Swedish | 1 | 221 | 195 |
| Total 9 | 7 | 22[a] | 1231 | 989 |

[a]One Swiss institution is represented in both the Swiss German and the Swiss Italian cultures.

**Table 2** Mood means according to culture (sorted by mean trial VI value)

| Country, Language | LASA anchors[a] | Trial VI *n* | Trial VI Mean[b] (95% CI) | Trial VII *n* | Trial VII Mean[b] (95% CI) |
|---|---|---|---|---|---|
| Slovenia, Slovenian | Srečna<br>Nesrečna | 130 | 54.6<br>(48.9, 60.0) | 87 | 68.4<br>(62.4, 74.0) |
| Sweden, Swedish | Lycklig<br>Olycklig | 221 | 62.9<br>(58.9, 66.6) | 194 | 66.5<br>(62.4, 70.4) |
| Switzerland/ Germany, German | Glücklich<br>Unglücklich | 196 | 70.8<br>(67.1, 74.3) | 155 | 71.8<br>(67.5, 75.7) |
| Spain, Spanish | Feliz<br>Desdichada | 49 | 71.7<br>(64.1, 78.4) | 20 | 76.9<br>(65.4, 86.1) |
| Italy, Italian | Buono<br>Pessimo | 156 | 74.2<br>(70.2, 77.9) | 162 | 73.9<br>(69.9, 77.6) |
| Switzerland, French | Bon<br>Mauvais | 63 | 74.1<br>(67.7, 79.8) | 52 | 82.0<br>(75.9, 87.2) |
| Australia/ New Zealand, English | Happy<br>Miserable | 309 | 77.9<br>(75.4, 80.4) | 184 | 81.6<br>(78.4, 84.5) |
| South Africa, English | Happy<br>Miserable | 43 | 79.3<br>(72.2, 85.3) | 49 | 81.7<br>(75.3, 87.1) |
| Switzerland, Italian | Buono<br>Pessimo | 42 | 83.8<br>(77.4, 89.1) | 57 | 84.4<br>(79.0, 89.1) |

[a]Patients were asked to mark a cross on a 100-mm line to rate mood overall for the entire period since their last full clinical assessment. Higher scores reflect better mood. [b]Transformed mean as described in Statistical methods.

© Cancer Research Campaign 1998

Table 3 Summary of the impact of biomedical factors on baseline QL measures in trial VI and VII (two-sided P-values)

| | Emotional wellbeing (Bf-S) | | Mood | | Physical wellbeing | | Appetite | | Adjustment (PACIS) | |
|---|---|---|---|---|---|---|---|---|---|---|
| | VI | VII | VI | VII | VI | VII | VI | VII | VI | VII |
| *Non-tumour related factors*[a] | | | | | | | | | | |
| Age: 60 + (VII) | | 0.002 | | 0.002 | | | | | | 0.0009 |
| Chronic disease: no | | | | | | 0.02 | | | | |
| Treatment assignment: less chemotherapy | | | | | | | 0.007 | | | |
| *Tumour related factors*[a] | | | | | | | | | | |
| ER status: positive | | | 0.0005 | | | | | | | |
| Progesterone receptor status | | | | | | | | | | |
| Number of positive nodes: fewer positive nodes | | 0.01 | 0.003 | 0.05 | | | | | 0.0008 | |
| Tumour size | | | | | | | | | | |
| Type of surgery: mastectomy | | | | 0.006 | | | | | | |

[a]Category of factor associated with higher QL scores.

Table 4 Emotional wellbeing, mood and adjustment means according to age in post-menopausal patients (trial VII)

| QL scale | Age | n | Mean[a] (95% CI) | P |
|---|---|---|---|---|
| Emotional wellbeing (Bf-S) | < 60 | 432 | 75.9 (73.9, 77.8) | 0.002 |
| | 60+ | 510 | 80.0 (78.3, 81.6) | |
| Mood | < 60 | 434 | 72.1 (69.6, 74.5) | 0.002 |
| | 60+ | 526 | 77.1 (75.0, 79.0) | |
| Adjustment (PACIS) | < 60 | 437 | 64.5 (61.7, 67.2) | 0.0009 |
| | 60+ | 532 | 70.5 (68.2, 72.7) | |

[a]Transformed mean as described in Statistical methods.

### Statistical methods

The LASA scales were scored by measuring in millimetres from 0 to 100, with higher numbers reflecting better QL, and the scores for the Bf-S were transformed to have the same range and interpretation. We used a transformation (the square root of 100 minus these scores) in all analyses, since this transformation approximated a normal distribution and stabilized the variances, but all results are reported in the 0 to 100 scale (100 minus the squares of the estimated means of the transformed scores). Because the square root transformation approximates a symmetric distribution, these 'transformed means' are approximate estimates of medians. Analyses of variance (ANOVA) were used to test for associations between baseline QL measures and various cultural, biomedical and sociodemographic factors. We previously showed that language had a significant influence on the QL scores obtained from our instruments (Hürny et al, 1992). Therefore, for this analysis we grouped patients into the nine language/country groups listed above and assessed each of the biomedical and sociodemographic factors controlling for culture but not for the other factors, fitting a separate ANOVA model for each of the five QL measures within each of the two trials. In addition, we investigated interactions between selected factors that we had found to be associated with baseline QL. We report transformed means standardized to the overall distribution of cultures within each trial. No adjustment was made for multiple comparisons; two-sided P-values were used as descriptive statistics to identify associations in the observed results.

As previously shown, baseline QL scores can vary with time from start of chemotherapy (Hürny et al, 1994). In this analysis,

© Cancer Research Campaign 1998

controlling for timing when testing the significance of culture did not change our conclusions. We did not control for timing when testing for biomedical and sociodemographic factors, because we expected that the effects of timing would be independent of these factors; overall, the proportion of the variance explained by timing was small.

### RESULTS

### Description of the sample

Baseline QL assessments were completed by 1262 (86%) of the 1475 trial VI patients and by 1008 (83%) of the 1212 trial VII patients. Compliance rates varied considerably among institutions, from 58% to 100% in trial VI and from 59% to 100% in trial VII. Excluded from this investigation were 50 patients from four countries who did not complete the QL form in the primary language of their region of their country (31 from trial VI, 19 from trial VII). Table 1 shows the culture groups, along with the number of institutions and the number of patients included in each.

### Cultural factors

Overall, cultural factors had the strongest impact on baseline QL and affected all five measures in both trials ($P < 0.0001$ for all, except for appetite and physical wellbeing in trial VII, which were $P = 0.05$ and 0.003 respectively). For example, Table 2 shows the means of the mood scale according to culture. The pattern of mood scores across culture groups was similar within the two trials.

Two of the languages were used in more than one country, allowing for comparisons between countries within the same language. QL scores for English-speaking patients from Australia/New Zealand were similar to those for English-speaking patients from South Africa (Table 2). In contrast, Italian-speaking patients from northern Italy reported substantially lower mood scores than those from the adjoining southern part of Switzerland ($P = 0.01$ in trial VI, $P = 0.002$ in trial VII; Table 2). All other QL scales showed differences in the same direction, although not all of the differences were statistically significant.

Overall, the variance explained by these cultural factors was modest. It ranged from 3% (Bf-S, physical wellbeing) to 8% (mood) in trial VI (premenopausal and perimenopausal patients), and from 2% (physical wellbeing, appetite) to 6% (mood, PACIS) in trial VII (post-menopausal patients).

### Biomedical factors

After controlling for cultural effects, biomedical factors also had an impact on the QL measures, as summarized in Table 3. The mood and adjustment scales were most responsive to these factors. The effects of tumour and non-tumour related biomedical factors were different in the two trials.

Age had a significant impact in post-menopausal patients only. Older patients (60+ years) reported better emotional wellbeing ($P = 0.002$) and mood ($P = 0.002$), and less effort to cope ($P = 0.0009$) compared with younger post-menopausal patients (Table 4). Although older patients were more likely to have total mastectomies (71% for patients under 55 years to 79% for patients 65 years and older), the age effect was independent of type of primary surgery.

Presence of concomitant chronic disease was associated with a tendency to worse physical wellbeing in post-menopausal patients

**Figure 1** Impact of tumour factors on mood scores in premenopausal patients. (**A**) Transformed mean mood score by ER status. (**B**) Transformed mean mood score by number of positive nodes

(no co-morbidity: mean = 79.7; with co-morbidity: mean = 76.2, $P = 0.02$). As expected, co-morbidity was more prevalent in post-menopausal (33%) than premenopausal (13%) patients. Type of surgery had an impact on mood in post-menopausal patients only. Those treated with mastectomy reported better mood scores than those treated with conservation plus radiotherapy (76.1 vs. 70.8, $P = 0.006$). An effect of systemic adjuvant treatment assignment was observed in trial VII. Patients assigned to receive tamoxifen only reported better appetite (mean = 88.3) than those who were to undergo additional early (mean = 82.7), late (mean = 83.6), or early and late chemotherapy (mean = 84.5, $P = 0.007$).

In general, patients whose tumours had characteristics associated with poorer prognoses reported worse QL. Premenopausal

© Cancer Research Campaign 1998

patients with ER-negative tumours reported worse mood than those with ER-positive tumours (67.2 vs. 73.0; $P = 0.0005$). Higher numbers of positive nodes were associated with worse mood in both premenopausal ($P = 0.003$) and post-menopausal patients ($P = 0.05$) and with worse emotional wellbeing in post-menopausal patients ($P = 0.01$); adjustment was negatively affected by status in premenopausal patients only ($P = 0.0008$). ER status and number of positive nodes were both independent predictors of mood in premenopausal patients ($P = 0.0004$ for ER status adjusted for number of positive nodes; $P = 0.002$ for number of positive nodes adjusted for ER status). The association with ER status was seen in eight of the nine cultural subgroups ($P \leq 0.05$ for French/Switzerland and Spain); the difference was small and not statistically significant in the one subgroup in which the direction of the difference was the opposite (Italy). Figure 1 shows mean mood scores according to ER status and number of positive nodes in all premenopausal patients.

### Sociodemographic factors

Sociodemographic factors were significantly associated with QL in post-menopausal, but not premenopausal, patients. Married and separated or divorced post-menopausal patients reported better emotional wellbeing (Bf-S) than those who were widowed or single (mean of married = 79.2, separated/divorced = 80.1, widowed = 75.3, single = 75.0; $P = 0.05$). Living alone was associated with poorer emotional wellbeing (mean = 75.3) than living with others (mean for women living with spouse or partner = 78.9, with children = 77.3, with other = 83.4; $P = 0.05$). Living alone or with children was associated with worse appetite than living with spouse, partner or other (mean for women living alone = 81.8, with children = 83.2, with spouse or partner = 85.8 with other = 88.4; $P = 0.05$). Post-menopausal women with either little formal schooling or with extensive education reported worse adjustment than women with levels in between (mean for 0–5 years = 67.1, 6 or 7 years = 66.5, 8 or 9 years = 69.0, 10–12 years = 70.6, more than 12 years = 59.4; $P = 0.04$).

### Country differences within language

We also investigated whether or not there was a differential impact of biomedical and sociodemographic factors on global QL indicators between different countries within the same language group. Mood and adjustment were analysed separately in the two English-speaking groups (South Africa and Australia/New Zealand) within each trial and in the two Italian-speaking groups (Italy and Switzerland) within each trial. Although some differences were observed, there were no consistent patterns, suggesting that these factors have a similar impact on patient self-estimation within language groups. They do not explain the overall difference in mood between Italian-speaking patients from Italy and Italian-speaking patients from Switzerland.

### DISCUSSION

Individual patients' baseline QL scores vary considerably within cancer clinical trials. They are highly predictive of the level of subsequent QL assessments and therefore relevant for investigating treatment-related changes. We analysed how biomedical, sociodemographic and cultural factors affected baseline scores of two large-scale adjuvant breast cancer trials (Hürny et al, 1996*a*) and now report these associations in more detail. This information will be useful for investigators in determining which additional variables need to be assessed in breast cancer clinical trials.

### Cultural factors

This investigation has confirmed and substantially extended our previous report (Hürny et al, 1992) that cultural and language factors affect patient-rated QL and, therefore, need to be considered in cross-cultural trials, although their contribution to the total variance in QL scores is modest. Although it remains possible that these findings are due to imperfect translation, we believe this is unlikely given the thorough multistep translation procedure which was used.

Before concluding that there are 'true' cultural differences, we should also consider institutional factors and the possibility of selective recruitment of patients with different characteristics, or differences in patient care in the various cultures. This may account for the differences seen between the Italian-speaking Swiss and the patients from northern Italy. The participating hospitals in Italy are larger, the waiting rooms are more crowded, and patients may feel more anonymous at the beginning of treatment than in the Swiss outpatient clinics. Also, the generally higher QL scores in Switzerland compared with Slovenia may reflect true overall socioeconomic differences between the two countries. It is also possible that systematic differences among cultures in the degree and method of doctor–patient communication about the details of the tumour and its treatment may underlie part of the observed cultural differences in QL.

On the other hand, in the case that patients from different cultures report similar QL scores (e.g. English-speaking patients from Australia/New Zealand and South Africa), this similarity may hide true cultural differences in perceptions, attitudes and habits. Although these differences are not relevant for overall treatment comparisons, in subgroup analyses an isolated interpretation of either symptoms or any aspect of QL without regard to the institutional, social and cultural context could be misleading (Hürny et al, 1993).

In summary, the overall differences among the investigated cultures probably reflect cultural differences in patients' response to disease and treatment sequelae, such as a tendency to stoic self-control or exasperated emotional expression in particular cultures. This is especially relevant for highly 'subjective' concepts such as 'coping' (PACIS), but was also seen in appetite, a relatively concrete sensation.

### Biomedical factors

Age had an effect on QL in post-menopausal patients only. Post-menopausal patients under 60 reported worse emotional and adjustment scores than older patients. To our knowledge, the role of age in the adjustment to diagnosis and treatment has not been reported separately within menopausal status groups (Vinokur et al, 1990; Ganz et al, 1993; Mor et al, 1994), except in one study in which increased anxiety was observed in patients in their fifties treated by mastectomy (Maraste et al, 1992).

Co-morbidity was much more frequent in post-menopausal patients. It had a weak negative impact on physical wellbeing in both trials, but was significantly associated with physical wellbeing only in post-menopausal patients.

© Cancer Research Campaign 1998

Extent of surgery was a significant factor associated with mood in post-menopausal patients, with patients who had had total mastectomies reporting better mood scores. At the start of adjuvant therapy, emotional distress is characterized by anxiety rather than depression (Maraste et al, 1992). A total mastectomy could have relieved anxiety regarding disease progression in these patients (Fallowfield et al, 1990), whereas in premenopausal patients other factors, such as concerns about body image, may dominate. Radiotherapy was given only after breast conservation, and followed chemotherapy. Its anticipation might have contributed to increased anxiety in the group treated by breast conservation. Other reports do not suggest a relationship between extent of surgery and either psychological distress or physical dysfunction at this early phase of recovery from surgery (Kiebert et al, 1991; Ganz et al, 1992; Pozo et al, 1992). The relationship between ER status and mood is in agreement with the finding reported by Razavi (1990), but has not been confirmed by other groups (Hislop and Kan, 1990; Maunsell and Brisson, 1990; Rosenqvist et al, 1993; Tjemsland et al, 1995). It may reflect either an intrinsic interaction between emotional processes and the endocrine system in premenopausal patients or the psychological impact of the physician having communicated to the patient information regarding her prognosis (Roberts et al, 1994). Interestingly, the association between ER status and perceived adjustment, which is not a pure emotional concept but has also a strong cognitive component, was not significant. On the other hand, the relationship between number of positive nodes and both emotional measures and adjustment may reflect patients' response to having received information regarding nodal involvement and its prognostic significance. The exact procedure and timing of patient information may have varied among and even within institutions. These issues cannot be further clarified with our data. Nevertheless, this finding is intriguing and warrants further study, especially in regard to treatment effects on QL and to biomedical outcome (Coates et al, 1992; Hürny, 1993).

The effect of treatment assignment on appetite in post-menopausal patients is probably an anticipatory effect. Patients were informed about the allocated treatment before completing the QL form, patients assigned to receive tamoxifen only reported better baseline appetite scores than those assigned to receive late chemotherapy only. As reported in the analysis of the impact of treatment on subsequent QL in these trials (Hürny et al, 1996*a*), anticipation of treatment plays a role in patients' perception of QL.

Overall, biomedical factors showed little impact on baseline QL. Because treatment trials are stratified by major biomedical prognostic factors, they are primarily relevant for subgroup comparisons. Our findings contrast with those of a smaller investigation of 109 newly diagnosed breast cancer patients, which found no significant association between patient-rated rehabilitation needs and axillary node status, type of surgery or receipt of chemotherapy (Ganz et al, 1990). It is not clear whether this difference is due to the different QL measures (global indicators vs specific problem evaluation) or due to the different study contexts. In our study, mood and adjustment, those measures most closely reflecting patients' 'subjective' experience, were the most sensitive to biomedical factors.

### Sociodemographic factors

Sociodemographic factors showed a relatively weak association with baseline QL, which was seen in post-menopausal patients only. Overall, we found no major effect of these factors on QL. However, these factors have been shown to predict survival in breast (Karjalainen and Pukkala, 1990; Schrijvers et al, 1995) and other cancers, and we suggest that in large-scale international trials at least one indicator of socioeconomic status (e.g. educational level) be recorded.

### Further investigations

More information is needed about the perception and understanding of cancer and its treatment in various cultures. The question of how interactions among cultural, social and biomedical factors affect QL in cancer clinical trial settings warrants further study, not just for baseline scores, but also for QL during treatment and follow-up. In addition, the generalizability of the effect of biomedical interventions on QL should be investigated within international clinical trials.

Only a modest part of the variance in QL scores was explained by the factors investigated. In contrast, individual psychosocial factors, such as stressful life events before diagnosis (Maunsell et al, 1992), history of depression (Maunsell et al, 1992), attitudes (Carver et al, 1994) and social support (Irvine et al, 1991) have been reported to be substantially associated with patients' adjustment. Their impact as covariates in evaluating treatment effects on QL could not be investigated in the present study.

### Conclusions

Cultural factors have a substantial impact on baseline QL measures, although they explain only a small percentage of the total variance. They need to be considered when evaluating the influence of treatment on QL in international breast cancer clinical trials. Various biomedical factors have a less pronounced but tangible impact. Their effects are different in premenopausal compared with post-menopausal patients.

### ABBREVIATIONS

ANOVA, analysis of variance; Bf-S, Befindlichkeitsskala; CMF, combination chemotherapy using oral cyclophosphamide plus intravenous methotrexate and 5-fluorouracil; ER, oestrogen receptor; IBCSG, International Breast Cancer Study Group; LASA, linear analogue self-assessment; PACIS, perceived adjustment to chronic illness scale; QL, quality of life

### ACKNOWLEDGEMENTS

We gratefully acknowledge the support for central trial coordination, data management, and statistics provided by the Swiss Cancer League, Cancer League of Ticino, Swiss Group for Clinical Cancer Research, Australian–New Zealand Breast Cancer Trials Group, the Australian Cancer Society, Frontier Science and Technology Research Foundation, and grant PBR-53 from the American Cancer Society. The trials were also supported by the National Health and Medical Research Council of Australia, the Anti-Cancer Council of Victoria and the New South Wales Cancer Council. These data reflect the efforts of our patients, nurses, data managers and physicians. Without their enthusiastic cooperation across disciplines, national borders and cultures, studying quality of life in international cancer clinical trials would not be possible. We are especially grateful to Mary Isley, Rita Hinkle and Heidi

© Cancer Research Campaign 1998

Gusset, who were responsible for central QL data management. We also would like to thank Christoph Minder for his contribution to the analysis of cultural and sociodemographic factors.

### REFERENCES

Bernhard J, Hürny C, Coates AS and Gelber RD (1996) Applying quality of life principles in international cancer clinical trials. In *Quality of Life and Pharmacoeconomics in Clinical Trials*. B Spilker (ed), pp. 693–705. Lippincott-Raven: Philadelphia

Butow PN, Coates AS, Dunn S, Bernhard J and Hürny C (1991) On the receiving end. IV: Validation of quality of life indicators. *Ann Oncol* **2**: 597–603

Carver CS, Pozo Kaderman C, Harris SD, Noriega V, Scheier MF, Robinson DS, Ketcham AS, Moffat Jr FL and Clark KC (1994) Optimism versus pessimism predicts the quality of women's adjustment to early stage breast cancer. *Cancer* **73**: 1213–1220

Cella DF, Lloyd S and Wright B (1996) Cross-cultural instrument equating: current research and future directions. In *Quality of Life and Pharmacoeconomics in Clinical Trials*. B Spilker (ed), pp. 707–715. Lippincott-Raven: Philadelphia

Coates AS, Fischer-Dillenbeck C, McNeil DR, Kaye SB, Sims K, Fox RM, Hedley DW, Raghavan DR, Woods RL, Milton GW and Tattersall MHN (1983) On the receiving end. II. Linear analogue self assessment in the evaluation of quality of life of cancer patients. *Eur J Cancer Clin Oncol* **19**: 1633–1638

Coates AS, Gebski V, Bishop JF, Jeal PN, Woods RL, Snyder R, Tattersall MHN, Byrne M, Harvey V, Gill PG, Simpson J, Drummond R, Browne J, Van Cooten R and Forbes JF (1987) Improving the quality of life in advanced breast cancer. A comparison of continuous and intermittent treatment strategies. *N Engl J Med* **317**: 1490–1495

Coates AS, Gebski V, Signorini D, Murray P, McNeil D, Byrne M and Forbes JF (1992) Prognostic value of quality-of-life scores during chemotherapy for advanced breast cancer. Australian New Zealand Breast Cancer Trials Group [see comments]. *J Clin Oncol* **10**: 1833–1838

Coates AS, Glasziou PP and McNeil D (1990) On the receiving end. III: Measurement of quality of life during cancer chemotherapy. *Ann Oncol* **1**: 213–217

Fallowfield LJ, Hall A, Maguire GP and Baum M (1990) Psychological outcomes of different treatment policies in women with early breast cancer outside a clinical trial. *Br Med J* **301**: 575–580

Ganz PA, Hirji K, Sim MS, Schag CA, Fred C and Polinsky ML (1993) Predicting psychosocial risk in patients with breast cancer. *Med Care* **31**: 419–431

Ganz PA, Schag AC, Lee JJ, Polinsky ML and Tan SJ (1992) Breast conservation versus mastectomy. Is there a difference in psychological adjustment or quality of life in the year after surgery? *Cancer* **69**: 1729–1738

Ganz PA, Schag CA and Cheng HL (1990) Assessing the quality of life – a study in newly-diagnosed breast cancer patients. *J Clin Epidemiol* **43**: 75–86

Hislop TG and Kan L (1990) Receptor status and psychological adjustment of breast cancer patients (letter). *Lancet* **336**: 47–48

Hürny C (1993) Coping and survival in early breast cancer: an update. *Recent Results Cancer Res* **127**: 211–220

Hürny C, Bernhard J, Bacchi M, van Wegberg B, Tomamichel M, Spek U, Coates AS, Castiglione M, Goldhirsch A, Senn H-J and for the SAKK and IBCSG (1993) The Perceived Adjustment to Chronic Illness Scale (PACIS): a global indicator of coping for operable breast cancer patients in clinical trials. *Supportive Care Cancer* **1**: 200–208

Hürny C, Bernhard J, Coates AS, Castiglione M, Peterson H, Gelber RD and for the IBCSG (1994) Timing of baseline quality of life assessment in an international adjuvant breast cancer trial: its effect on patient self-estimation. *Ann Oncol* **5**: 65–74

Hürny C, Bernhard J, Coates AS, Castiglione M, Peterson HF, Gelber RD, Forbes JF, Rudenstam C-M, Simoncini E, Crivellari D, Goldhirsch A and Senn H-J (1996*a*) Impact of adjuvant therapy on quality of life in women with node-positive operable breast cancer. *Lancet* **347**: 1279–1284

Hürny C, Bernhard J, Coates AS, Peterson HF, Castiglione-Gertsch M, Gelber RD, Rudenstam C-M, Collins J, Lindtner J, Goldhirsch A and Senn H-J (1996*b*) Responsiveness of a single-item indicator versus a multi-item scale: assessment of emotional wellbeing in an international breast cancer trial. *Med Care* **34**: 234–248

Hürny C, Bernhard J, Gelber RD, Coates AS, Castiglione M, Isley M, Dreher D, Peterson H, Goldhirsch A and Senn H-J (1992) Quality of life measures for patients receiving adjuvant therapy for breast cancer: an international trial. International Breast Cancer Study Group. *Eur J Cancer* **28**: 118–124

International Breast Cancer Study Group (1996) Duration and reintroduction of adjuvant chemotherapy for node-positive premenopausal breast cancer patients. *J Clin Oncol* **14**: 1885–1893

International Breast Cancer Study Group (1997) Effectiveness of adjuvant chemotherapy in combination with tamoxifen for node-positive post-menopausal breast cancer patients. *J Clin Oncol* **15**: 1385–1394

Irvine D, Brown B, Crooks D, Roberts J and Browne G (1991) Psychosocial adjustment in women with breast cancer. *Cancer* **67**: 1097–1117

Karjalainen S and Pukkala E (1990) Social class as a prognostic factor in breast cancer survival. *Cancer* **66**: 819–826

Kiebert GM, de Haes JC and van de Velde CJ (1991) The impact of breast-conserving treatment and mastectomy on the quality of life of early-stage breast cancer patients: a review. *J Clin Oncol* **9**: 1059–1070

Maraste R, Brandt L, Olsson H and Ryde Brandt B (1992) Anxiety and depression in breast cancer patients at start of adjuvant radiotherapy. Relations to age and type of surgery. *Acta Oncol* **31**: 641–643

Maunsell E and Brisson J (1990) Receptor status and psychological adjustment of breast cancer patients (letter). *Lancet* **336**: 47

Maunsell E, Brisson J and Deschenes L (1992) Psychological distress after initial treatment of breast cancer. Assessment of potential risk factors. *Cancer* **70**: 120–125

Mor V, Malin M and Allen S (1994) Age differences in the psychosocial problems encountered by breast cancer patients. *Monogr Natl Cancer Inst* 191–197

Pozo C, Carver CS, Noriega V, Harris SD, Robinson DS, Ketcham AS, Legaspi A, Moffat Jr FL and Clark KC (1992) Effects of mastectomy versus lumpectomy on emotional adjustment to breast cancer: a prospective study of the first year postsurgery. *J Clin Oncol* **10**: 1292–1298

Priestman TJ and Baum M (1976) Evaluation of quality of life in patients receiving treatment for advanced breast cancer. *Lancet* **i**: 899–901

Razavi D, Farvacques C and Delvaux N (1990) Psychosocial correlates of oestrogen and progesterone receptors in breast cancer. *Lancet* **335**: 931–933

Roberts CS, Cox CE, Reintgen DS, Baile WF and Gibertini M (1994) Influence of physician communication on newly diagnosed breast patients' psychologic adjustment and decision-making. *Cancer* **74**: 336–341

Rosenqvist S, Berglund G, Bolund C, Fornander T, Rutqvist LE, Skoog L and Wilking N (1993) Lack of correlation between anxiety parameters and oestrogen receptor status in early breast cancer. *Eur J Cancer* **29A**: 1325–1326

Schrijvers CT, Mackenbach JP, Lutz JM, Quinn MJ and Coleman MP (1995) Deprivation and survival from breast cancer. *Br J Cancer* **72**: 738–743

Tjemsland L, Soreide JA and Malt UF (1995) Psychosocial factors in women with operable breast cancer. An association to estrogen receptor status? *J Psychosom Res* **39**: 875–881

Vinokur AD, Threatt BA, Vinokur Kaplan D and Satariano WA (1990) The process of recovery from breast cancer for younger and older patients. Changes during the first year. *Cancer* **65**: 1242–1254

von Zerssen D (1976) *Klinische Selbstbeurteilungsskalen (KSb-S) aus dem Muenchener Psychiatrischen Informationssystem (PSYCHIS Muenchen). Die Befindlichkeitsskala-Parallelformen Bf-S und Bf-S'. Manual*. Beltz: Weinheim von Zerssen D (1986) Clinical self-rating scales (CSRS) of the Munich Psychiatric Information System (PSYCHIS Muenchen). In *Assessment of Depression*. N Sartorius and TA Ban (eds), pp. 270–303. Springer: Berlin

### APPENDIX

### International Breast Cancer Study Group: participants and authors trials VI, VII

| | |
|---|---|
| Coordinating Center<br>Bern, Switzerland | M Castiglione, A Goldhirsch (Studies Coordinators), K Geiser, A Berlinger, G Egli (Data-Management) R Maibach, R Pedowski |
| Statistical Center<br>Harvard School of Public Health and Dana-Farber Cancer Institute, Boston, MA, USA | R Gelber (Group Statistician), K Price, H Peterson, M Zelen, S Gelber, A O'Neill |
| Quality of Life Office<br>Bern, Switzerland | J Bernhard, Ch Hürny, H Gusset |
| Pathology Office<br>Institute of Cancer Research, Royal Cancer Hospital, Sutton, UK | B Gusterson, R Bettelheim, R Reed |
| Data Management Center<br>Frontier Science and Technology Research Foundation Amherst, NY, USA | M Isley, R Hinkle |

© Cancer Research Campaign 1998

| | |
|---|---|
| Auckland Breast Cancer Study Group, Auckland, New Zealand | RG Kay, VJ Harvey, CS Benjamin, P Thompson, A Bierre, M Miller, B Hochstein, A Lethaby, J Webber |
| Centro di Riferimento Oncologico Aviano, Italy | D Crivellari, S Monfardini, E Galligioni, A Veronesi, A Buonadonna, S Massarut, C Rossi, E Candiani, A Carbone, R Volpe, M Roncadin, M Arcicasa, GF Santini, F Villalta, F Coran, S Morassut |
| Spedali Civili & Fondazione Beretta, Brescia, Italy | G Marini, E Simoncini, P Marpicati, A Barni, P Grigolato, L Morassi |
| Groote Schuur Hospital, Cape Town, Rep. of South Africa | DM Dent, A Gudgeon, E Murray, P Steynor, J Toop |
| West Swedish Breast Cancer Study Group, Göteborg, Sweden | CM Rudenstam, A Wallgren, S Ottosson-Lönn, R Hultborn, G Colldahl-Jäderström, E Cahlin, J Mattsson, S Holmberg, S Jansson, L Ivarsson, O Ruusvik, LG Niklasson, S Dahlin, G Karlsson, B Lindberg, A Sundbäck, S Bergegårdh, H Salander, C Andersson, M Heideman, Y Hessman, O Nelzén, G Claes, T Ramhult, JH Svensson, P Liedberg |
| General Hospital Gorizia, Italy | S Foladore, L Foghin, G Pamich, C Bianchi, B Marino, A Murgia, V Milan |
| Sandton Oncology Center Johannesburg, South Africa | D Vorobiof, M Chasen, G Fotheringham, G de Muelenaere, B Skudowitz, C Mohammed, A Rosengarten |
| The institute of Oncology, Ljubljana, Slovenia | J Lindtner, D Erzen, E Majdic, B Stabuc, A Plesnicar, R Golouh, J Lamovec, J Jancar, I Vrhoved, M Kramberger |
| Madrid Breast Cancer Group, Madrid, Spain | H Cortés-Funes, D Mendiola, C Gravalos, Colomer, M Mendez, F Cruz Vigo, P Miranda, A Sierra, F Martinez-Tello, A Garzon, S Alonso, A Ferrero, C Vargas |
| Anti-Cancer Council of Victoria, Melbourne, Australia | J Collins, P Gregory, P Kitchen, S Hart, S Neil, M Henderson, I Russell, T Gale, M Pitcher, R Snyder, R McLennan, M Schwarz, I Burns, M Green, R Basser, R Drummond, A Rodger, G Richardson, J McKendrick, M Chipman |
| Royal Adelaide Hospital, Adelaide, Australia | I Olver, A Robertson, P Gill, ML Carter, P Malycha, E Yeoh, G Ward, ASY Leong, J Lommax-Smith, D Hoosfall, R D'Angelo |
| Sir Charles Gairdner Hospital, Nedlands, Western Australia | M Byrne, G van Hazel, J Dewar, M Buck, D Ingram, G Sterret |
| Australian New Zealand Breast Cancer Trials Group (ANZ BCTG) Mater Hospital Waratah, Newcastle, Australia | JF Forbes, J Stewart, D Jackson, R Gourlay, J Bishop, C Flower, A Wilson, S Cox, S Ackland, A Bonaventura, C Hamilton, J Denham, P O'Brien, M Back, S Brae, A Price, Muragasu, H Foster, D Clarke, R Sillar, V Clarke, S Brew |
| University of Sydney, Dubbo Base Hospital and Royal Prince Alfred Hospital, Sydney, Australia | MHN Tattersall, A Coates, F Niesche, R West, S Renwick, J Donovan, P Duval, R J Simes, A Ng, D Glenn, RA North, J Beith, RG O'Connor, M Rice, G Stevens, J Grassby, S Pendlebury, C McLeod, M Boyer, A Sullivan, J Hobbs |
| European Institute of Oncology, Milan, Italy | A Goldhirsch, G Martinelli, U Veronesi, A Luini, R Orecchia, G Viale, M Colleoni, F Nolè, F Peccatori, A Costa, S Zurrida, P Veronesi, V Sacchini, V Gallimberti |
| Ospedale Infermi, Rimini | A Ravaioli, D Tassinari, G Oliverio, F Barbanti, P Rinaldi, E Pini, G Drudi |
| Ospedale S Eugenio, Rome, Italy | M Antimi, M Minelli, V Bellini, R Porzio, E Pernazza, G Santeusanio, LG Spagnoli |
| Ospedale S Bortolo, Vicenza, Italy | M Magazu, V Fosser, P Morandi, G Scalco, M Balli, M Gion, S Meli, G Torsello |
| Toronto Sunnybrook Regional Cancer Centre Toronto, Canada | K Pritchard, D Sutherland, C Sawka, G Taylor, R Choo, C Catzavelos, K Roche |
| SAKK (Swiss Group for Clinical Cancer Research): | |
| Inselspital, Bern | MF Fey, E Dreher, H Schneider, S Aebi, K Buser, J Ludin, G Beck, H Bürgi, A Haenel, JM Luthi, R Markwalder, HJ Altermatt, M Nandedkar |
| Kantonsspital, St. Gallen | HJ Senn, B Thürlimann, Ch Oehlschlegel, G Ries, M Töpfer, U Lorenz, O Schiltknecht, B Späti |
| Ospedale San Giovanni, Bellinzona | F Cavalli, O Pagani, H Neuenschwander, W Müller, L Bronz, C Sessa, G Martinelli, M Ghielmini, P Luscieti, E Passega, T Rusca, P Rey, J Bernier, S Martinoli, E Pedrinis, G Losa, T Gyr, L Leidi, G Pastorelli, A Goldhirsch |
| Kantonsspital, Basel | R Hermann, JF Harder, O Köchli, U Eppenberger, J Torhorst |
| Hôpital des Cadolles, Neuchâtel | D Piguet, P Siegenthaler, V Barrelet, RP Baumann |
| Kantonsspital, Luzern | R Joss |
| Kantonsspital, Zürich | B Pestalozzi, C Sauter, V Engeler, U Haller, U Metzger, P Huguenin, R Caduff |
| Centre Hôpitalier Universitaire, Lausanne | L Perey, S Leyvraz, P Anani, F Gomez, D Wellman, G Chapuis, P De Grandi, P Reymond, M Gillet, JF Delaloye |
| Hôpital Cantonal, Geneva | P Alberto, H Bonnefoi, P Schäfer, F Krauer, M Forni, M Aapro, R Egeli, R Megevand, E Jacot-des-Combes, A Schindler, B Borisch, S Diebold |
| Kantonsspital Graubünden, Chur | F Egli, P Forrer, A Willi, R Steiner, J Allemann, T Rüedi, A Leutenegger, U Dalla Torre |
| Swiss Cancer League, Bern, Switzerland | U Metzger, W Weber, G Noseda |

© Cancer Research Campaign 1998